# Sleep Architecture and Risk-Taking in Adolescents and Young Adults: Independent Contributions of REM Sleep, Sigma Activity, and Morning Cortisol †

**DOI:** 10.3390/bs16091611

**Published:** 2026-09-09

**Authors:** Laura B. F. Kurdziel, Autumn R. Vance, Abigail K. Dunn, Manikandan Santhanaraman, Amanda Cremone-Caira, Melissa A. St. Hilaire

**Affiliations:** 1 Department of Psychology and Neuroscience, Merrimack College, North Andover, MA 01845, USA; cremonea@merrimack.edu; 2 Department of Natural Sciences, Merrimack College, North Andover, MA 01845, USA; vancea@merrimack.edu (A.R.V.); dunnak@merrimack.edu (A.K.D.); 3 Shared Instrumentation and Research Facility, Merrimack College, North Andover, MA 01845, USA; santhanaramm@merrimack.edu; 4 Department of Computer and Data Science, Merrimack College, North Andover, MA 01845, USA; sthilairem@merrimack.edu

**Keywords:** sleep, adolescents, risk-taking, cortisol awakening response

## Abstract

Insufficient sleep is highly prevalent among adolescents and young adults and has well-documented effects on neural systems underlying decision-making and risk-taking behavior. While prior research has linked sleep deprivation to poorer decision-making broadly, less is known about how specific features of sleep architecture contribute to risk-taking behavior, or whether the cortisol awakening response (CAR), shaped in part by the preceding night’s sleep, represents an independent hormonal pathway linking sleep physiology to morning behavioral outcomes. The present study used a cross-sectional design to examine associations among objective sleep architecture, the CAR, and next-morning risk-taking behavior in a community sample of adolescents and young adults. We predicted that (1) greater rapid eye movement (REM) sleep percentage would be associated with better overall task performance, and (2) greater N2-N3 sigma power would be associated with reduced risk-taking under uncertainty. The role of the CAR was examined exploratorily. Fifty-five adolescents and young adults (aged 15–34; mean age = 18.9 years, *SD* = 3.08 years, 86% female) completed overnight sleep monitoring using a wearable EEG device, provided morning saliva samples to index the CAR (*n* = 28), and completed the Balloon Emotional Learning Task (BELT) to assess risk-taking behavior. Primary sleep variables of interest included REM sleep percentage and Non-REM sleep (N2-N3) sigma power. Behavioral outcomes included overall task performance and risk-taking under uncertainty. Linear regression models with age as a covariate were used for all primary analyses. Higher REM sleep percentage was associated with greater overall task performance, while higher N2-N3 sigma power was associated with reduced risk-taking under uncertainty, consistent with predictions. Greater CAR was independently associated with poorer task performance but did not moderate sleep-behavior associations, nor was it significantly associated with objective sleep measures. These findings highlight the stage-specific contributions of sleep architecture to next-morning risk-taking behavior in adolescents and young adults, with sleep physiology and cortisol responsivity emerging as distinct physiological contributors to behavioral outcomes.

## 1. Introduction

Adequate sleep is foundational to the neural processes that support decision-making and healthy risk-taking behavior, yet insufficient sleep has become increasingly prevalent across the lifespan. The National Sleep Foundation recommends 8–10 h of sleep for adolescents and 7–9 h of sleep for young adults (Hirshkowitz et al., 2015). Data from the CDC’s Youth Risk Behavior Survey indicate that as of 2023, 77% of high school students were failing to obtain sufficient sleep per night (CDC, 2025). This pattern persists into young adulthood: estimates of the proportion of college students scoring above the clinical threshold for poor sleep on the Pittsburgh Sleep Quality Index (PSQI; Buysse et al., 1989) range from approximately 45% to 60%, varying with population and cutoff criterion (Becker et al., 2018; Schmickler et al., 2023). These deficits are driven in part by environmental and structural factors, such as early school and work start times, that work against the biological sleep needs of adolescents and young adults. Compounding this, puberty is associated with a circadian phase delay that pushes adolescents’ natural sleep and wake times later, exacerbating misalignment between environmental pressures and biological rhythms (Crowley et al., 2018).

The consequences of insufficient sleep are wide-ranging, encompassing poorer mental health outcomes such as increased anxiety (Talbot et al., 2010; Uccella et al., 2023), depressive symptoms and lower self-esteem (Fredriksen et al., 2004; Uccella et al., 2023), as well as behavioral changes including greater impulsivity (Anderson & Platten, 2011) and elevated unhealthy risk-taking behaviors including substance use and other adverse outcomes (O’Brien & Mindell, 2005; Johri et al., 2025). It is worth noting that risk-taking itself is not inherently maladaptive. During adolescence, a degree of risk-taking serves important developmental functions, supporting exploration, identity formation, and the acquisition of experience necessary for adult independence (Romer et al., 2017; Steinberg, 2008). The concern, therefore, is not risk-taking per se, but rather the disruption of the regulatory systems that allow adolescents to calibrate risk adaptively. Given the prevalence of insufficient sleep across adolescence and young adulthood, it is necessary to understand the specific neural and physiological mechanisms through which sleep shapes risk-taking and risky decision-making behavior during this developmental period.

Although adolescence is the primary period of interest, the present study enrolled participants through age 34, reflecting the fact that prefrontal maturation and its regulatory influence over reward-driven behavior continues into the mid-20s (Caballero et al., 2016; Steinberg, 2008). Further, the transition from adolescence to adulthood represents a continuous developmental shift, consistent with an emerging adulthood framework (Arnett, 2000), during which risk-taking behavior remains elevated relative to later life (Romer et al., 2017). This framing is consistent with arguments for a life-span perspective on risk-taking, which note that increases in mortality and morbidity from childhood to adolescence are modest and continue rising into young adulthood, casting doubt on accounts that treat adolescence as uniquely vulnerable (Willoughby et al., 2014). Such perspectives also emphasize that risky behavior is not invariably impulsive or unregulated, and may instead reflect deliberate choice under conditions of uncertainty, which is relevant to the present focus on how physiological state relates to the calibration of risk rather than to risk propensity alone.

Of particular relevance are two distinct aspects of sleep physiology, rapid eye movement (REM) sleep and sigma band activity during Non-REM sleep, each of which shapes risky decision-making through separate but complementary mechanisms. REM sleep is characterized by heightened activity in regions central to emotional processing and decision-making, including the insula, medial prefrontal cortex, and the amygdala (see review: Goldstein & Walker, 2014). The prefrontal cortex is highly susceptible to sleep deprivation, and REM sleep deprivation in particular. Given its central role in executive functioning, sleep loss predictably impairs decision-making and promotes riskier behavior choices (see review: Womack et al., 2013), and REM sleep specifically has been shown to be critical to decision-making under risky conditions, with experimentally reduced REM following partial sleep deprivation impairing Iowa Gambling Task performance in young adults (Brunet et al., 2020). REM sleep has further been shown to support implicit learning in young adults, both for probabilistic classification in the weather prediction task following a daytime nap (Barsky et al., 2015) and for the transfer of implicit procedural knowledge (Brand et al., 2010). Sleep deprivation also appears to impair the ability to learn from the consequences of risky decisions, a process linked to activity in the dorsal anterior cingulate and the right nucleus accumbens, regions involved in error monitoring and reward anticipation, respectively (Venkatraman et al., 2007). Together, the insula, the dorsal anterior cingulate cortex, and the amygdala form the core nodes of the salience network (Uddin, 2016), a system that integrates emotional and interoceptive signals to guide behavioral responses (Chong et al., 2017; Jenkins et al., 2017; Schimmelpfennig et al., 2023). The insula in particular has been shown to exert an emotional influence on risky decision-making (see review: Uddin et al., 2017), suggesting that the activation of these regions during REM sleep may be directly relevant to how individuals evaluate and respond to risk. Taken together, these findings suggest that REM sleep plays a meaningful role in the neural regulation of risk-taking behavior, though it is not the only stage of sleep with implications for decision-making.

Sleep spindles, which are bursts of oscillatory activity occurring during NREM sleep (Lüthi, 2014; Schönauer & Pöhlchen, 2018), serve as a second, distinct mechanism through which sleep architecture shapes cognition. Sleep spindles facilitate hippocampal-prefrontal communication that supports higher-order cognitive functions, such as memory consolidation (Fogel & Smith, 2011; Schabus et al., 2004), learning (Schabus et al., 2006), planning, and decision-making (Morales-Ghinaglia et al., 2025; Vermeulen et al., 2019). Greater frontal spindle density, or the frequency of spindles observed, has been associated with faster response times on a risk-based decision-making task, suggesting that spindle activity may index the efficiency of neural communication underlying those decisions (Mark Lawrence et al., 2020). Higher sleep spindle amplitude during NREM sleep has been positively and independently associated with reasoning ability, suggesting that spindles serve as a biological marker of the cognitive processes relevant to flexible, logic-based decision-making (Fang et al., 2019).

While prior research has examined discrete spindle detection metrics such as spindle density, the present study used spectral power in the sigma frequency range (12–16 Hz) as a proxy for sleep spindle activity, consistent with established practice in sleep neurophysiological research (Campbell & Feinberg, 2016; Fogel & Smith, 2011; McClain et al., 2016; Mölle et al., 2002). It is important to note, however, that although sleep spindles and sigma-band power are closely related, they are not mathematically equivalent. Spindle detection yields discrete event-level parameters (density, amplitude, duration), whereas sigma power reflects the aggregate spectral energy in the 12–16 Hz range and is therefore jointly determined by these parameters as well as by non-spindle activity in the same band. Our rationale for this measure, and its limits, is detailed in Section 2.3.1. In our study, we hypothesize that sigma power during NREM stage 2 and stage 3 sleep (N2-N3 sigma) may reflect the degree to which prefrontal-hippocampal consolidation occurred overnight, with downstream consequences for how participants navigate risk and uncertainty on a behavioral task the following morning.

Together, REM sleep and NREM sigma-band activity represent two complementary pathways through which sleep architecture shapes the neural systems underlying emotional regulation, learning, and adaptive decision-making. Disruptions to these sleep stages therefore carry consequences that extend beyond daytime fatigue or inattention, directly undermining the prefrontal cortical and limbic systems that support sound judgement and behavioral regulation. This is particularly relevant for adolescents and young adults, a population already navigating consequential decisions around health, relationships, and academics, whose sleep is simultaneously greatly disrupted by biological and environmental pressures (Carskadon, 2011; Crowley et al., 2007, 2018). When the neural systems that support sound decision-making are compromised by sleep loss during the critical developmental window, the stakes extend well beyond performance on a laboratory task.

The influence of sleep on next-morning behavior is not limited to the neural consolidation that occurs during REM and NREM sleep—the architecture of sleep also regulates the hormonal environment upon awakening (Kim et al., 2015). Specifically, the pattern of sleep stages across the night shapes activity of the hypothalamic–pituitary–adrenal (HPA) axis, which governs the release of cortisol upon waking and may provide an additional physiological pathway through which sleep quality influences morning cognition and behavior (Backhaus et al., 2004; Devine & Wolf, 2016). Cortisol plays a broad regulatory role in arousal, attention, and cognitive readiness (Xiong et al., 2025). At moderate levels, cortisol facilitates alertness, decision-making, and executive function (Duque et al., 2022; Xiong et al., 2021; Zeng et al., 2024), while dysregulated cortisol patterns, including chronically elevated or blunted output, flattened diurnal rhythms, and altered cortisol awakening responses, have been associated with altered emotional processing and disrupted decision-making (George et al., 2025). The morning cortisol surge, known as the cortisol awakening response (CAR), is thought to prepare the organism for the cognitive and physical demands of the upcoming day, mobilizing energy resources and priming attentional systems (Fries et al., 2009; Stalder et al., 2016; Xiong et al., 2025).

The CAR is characterized by a rapid rise in free cortisol levels in the 30–45 min following morning awakening (Pruessner et al., 1997). Critically, this cortisol rise is specific to morning awakening in that it is not observed when individuals are woken during earlier stages of the night, suggesting a strong circadian component to the response (Bowles et al., 2022; Dettenborn et al., 2007), although the CAR does require awakening from sleep which is independent of circadian influences (Vargas & Lopez-Duran, 2020; Wilhelm et al., 2007). This specificity appears to reflect the inhibitory effect of slow-wave sleep on the HPA axis, which suppresses cortisol secretion during early sleep, followed by HPA activation during REM and NREM stage 2 sleep in the latter half of the night (Born & Fehm, 1998). This sequence primes the cortisol surge at awakening. Following the CAR peak, cortisol levels decline progressively across the day, consistent with the circadian regulation of HPA axis activity (Stalder et al., 2022), reaching their nadir in the late evening and early hours of nocturnal sleep before rising again in the hours preceding awakening. Given that the CAR is shaped by the preceding night’s sleep architecture and circadian timing, with HPA activity suppressed during slow-wave sleep and rising as REM sleep predominates later in the night (Backhaus et al., 2004; Devine & Wolf, 2016), and with response magnitude varying by awakening time relative to circadian phase (Bowles et al., 2022), it presents a promising physiological pathway through which sleep may influence morning behavioral outcomes. This is particularly relevant for adolescent and young adult populations, who are especially vulnerable to disrupted sleep architecture and circadian misalignment.

The present study examines associations among sleep architecture, the cortisol awakening response, and risk-taking behavior in adolescents and young adults, a developmental population for whom sleep disruption is both biologically driven and environmentally compounded, and for whom the consequences of poor decision-making are particularly salient during this critical developmental window. By employing objective measures of sleep alongside behavioral and hormonal assessments, this study moves beyond self-reported sleep quality to examine the specific neural and endocrine mechanisms that may underlie individuals’ differences in risk-taking. Our study had two primary predictions (P1 and P2). Based on the established role of REM sleep in prefrontal regulation of the salience network and emotional decision-making, we predicted that (P1) greater REM sleep percentage would be associated with better overall performance on a behavioral risk-taking task. Additionally, given the role of Non-REM sigma activity in strengthening prefrontal connectivity and supporting top-down regulation of impulsive behavior, we predicted that (P2) greater sigma power would be associated with reduced risk-taking under uncertainty. With respect to the CAR, we explored whether it was associated with sleep architecture and whether it moderated the relationship between sleep physiology and risk-taking behavior, given evidence linking HPA axis activation during REM and stage 2 sleep to the cortisol rise at awakening. This analysis was exploratory and non-directional, as two opposing patterns were plausible: a robust CAR might be necessary for the regulatory benefits of sleep to be behaviorally expressed, or might instead compensate for poorer sleep. We therefore did not specify a direction in advance. Finally, to account for stable individual differences that may independently shape behavioral outcomes in this age group, personality measures of chronotype, reward and punishment sensitivity, and impulsivity were examined as potential correlates of sleep behavior, task performance, or endocrine responsivity.

## 2. Materials and Methods

### 2.1. Study Design and Variables

This study employed a cross-sectional design to examine associations among objective sleep physiology, hormonal stress responsivity, and next-morning risk-taking behavior in adolescents and young adults. The primary independent variables were REM sleep percentage and N2-N3 sigma-band spectral power (12–16 Hz), derived from overnight wearable EEG recording. The primary dependent variables were total points earned on the Balloon Emotional Learning Task (BELT; indexing overall task performance), total pumps in the variable condition (indexing risk-taking under uncertainty), and total number of pops (indexing maladaptive risk-taking). The cortisol awakening response (CAR), operationalized as AUCi across the 45-min post-awakening sampling window, was examined as a secondary independent variable in exploratory analyses. Age was included as a covariate in all primary models. Personality and chronotype measures (PSQI, MEQ, BIS/BAS, UPPS) were examined as exploratory correlates of sleep behavior, task performance, and endocrine responsivity. The details of the study are described below.

### 2.2. Participants

Participants were 55 individuals aged 15–34 years (*M* = 18.9, *SD* = 3.08), 86% of whom were female. Participants were recruited from the local community via flyers, social media, and word of mouth, as well as through the SONA participant recruitment system. Of the 55 participants, 45 completed the health and demographic surveys. The derivation of analytic subsamples is described in detail in Section 2.4 and Section 3.1; briefly, sleep analyses were conducted in *n* = 46 participants, behavioral task data were available for *n* = 50, and cortisol analyses were conducted in *n* = 28, with attrition in each case attributable to factors unrelated to study outcomes.

Among these participants, the majority identified as Caucasian (*n* = 33, 73.3%). Fourteen participants (31.1%) identified as Hispanic, Latinx, or of Spanish origin. Additionally, 6 participants (13.3%) identified as Black, 1 (2.2%) as Chinese, and 1 (2.2%) as Vietnamese, while 7 participants (15.6%) selected “Other (not listed).” Three participants (6.7%) reported more than one racial identity.

**Figure 1:**
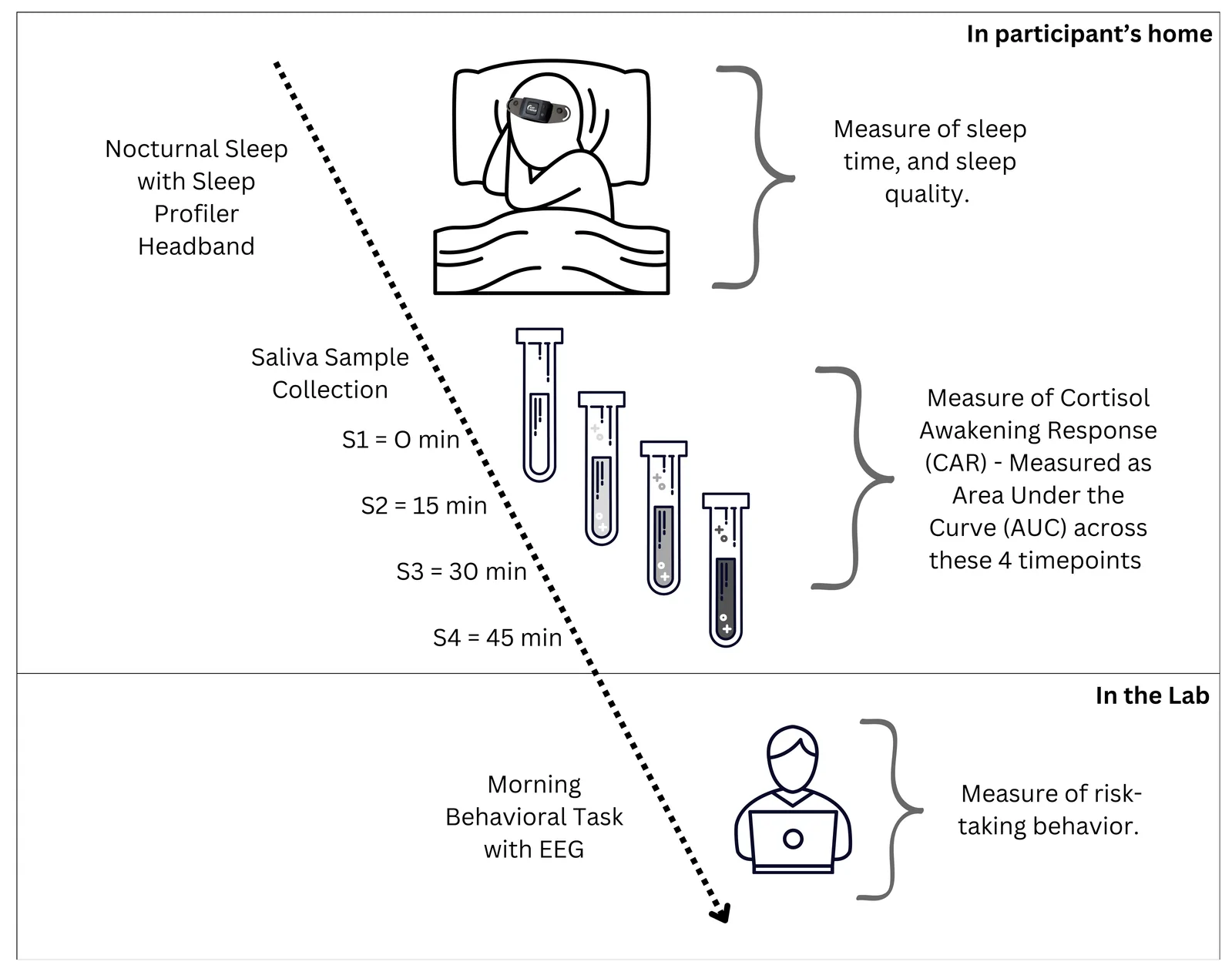
Study procedure timeline. Overnight sleep was recorded at home using a Sleep Profiler headband, followed by morning saliva sampling at 0, 15, 30, and 45 min to assess the cortisol awakening response (CAR). Participants then completed a laboratory-based behavioral task assessing risk-taking (BELT).

Participants completed an overnight sleep recording, morning saliva sampling, and an in-lab behavioral assessment (Figure 1). Exclusion criteria included color blindness (which would affect participants’ ability to perform the behavioral task), known sleep disorders, diseases or disorders that affect cortisol levels (e.g., Addison’s Disease, Cushing’s Syndrome, or another adrenal gland disorder), and medication use that could impact cortisol levels (e.g., corticosteroids, some antidepressants, anti-anxiety medications, antipsychotics, or stimulants). All participants provided informed consent in accordance with the college’s Institutional Review Board. For participants younger than 18 years, written parental consent and participant assent were obtained prior to participation.

### 2.3. Task and Procedures

#### 2.3.1. Sleep Physiology

Participants completed overnight sleep monitoring using the Sleep Profiler headband (Advanced Brain Monitoring, Carlsbad, CA, USA), a wearable EEG device that records from a frontopolar EEG montage rather than conventional derivations or dedicated EOG channels. Eye movements are detected from the frontopolar electrodes and incorporated into the automated staging algorithm. Automated sleep staging has been validated against simultaneous polysomnography with agreement comparable to inter-technologist reliability (Levendowski et al., 2017, 2023). Validation against simultaneous laboratory polysomnography demonstrated agreement between automated staging and five independent registered polysomnographic technologists (κ = 0.67), comparable to inter-technologist agreement (κ = 0.70), with agreement exceeding 80% for all sleep stages except N1 (Levendowski et al., 2017). Subsequent validation reported REM staging agreement of κ = 0.83 and κ = 0.72 across two independent recording sites (Levendowski et al., 2023). In addition to automated estimates of sleep stages, the device provides spectral power. These devices were used to derive measures of REM sleep and N2-N3 sigma activity.

We adopted sigma power rather than event-level spindle metrics for two reasons. First, spindle activity is topographically differentiated, with slow spindles predominating frontally and fast spindles centroparietally (Schabus et al., 2007; Cox et al., 2017). Our ambulatory frontopolar montage (Levendowski et al., 2017) therefore samples this activity differently from the central derivations on which spindle detection algorithms are typically developed. This is consequential because spindle inter-detector agreement is modest even under standard recording conditions (Warby et al., 2014; Lacourse et al., 2020), and is poorest for density and slow spindle parameters specifically (Ujma et al., 2015), the metrics most relevant to a frontal recording. Second, sigma power is continuous and highly stable within individuals (internight r ≈ 0.9; Tan et al., 2000), supporting its use as an index of trait-like differences. We therefore interpret findings at the level of sigma-band activity and do not attribute effects to specific spindle parameters. It is important to note that sigma power reported here reflects activity recorded at frontopolar derivations and should not be taken to index sigma activity across the scalp.

#### 2.3.2. Salivary Cortisol Collection

Salivary samples were collected using the passive drool method (Navazesh, 1993). Participants were instructed to gently allow their saliva into a tube through a provided straw and were explicitly told not to actively spit. They were asked to fill at least half of the tube, excluding any bubbles. Saliva was collected across four time points, at 0, 15, 30, and 45 min post-awakening, consistent with established protocols for indexing the CAR (Stalder et al., 2016). Participants were instructed to refrain from eating, drinking, exercising, and brushing their teeth prior to and during the saliva collection window. However, caffeine and nicotine consumption were not restricted or recorded across the study period, and menstrual phase and hormonal contraceptive use were not assessed; the implications of this are discussed in Section 4.2. Completed saliva tubes were returned to the laboratory within 12 h of collection, where they were stored at −80 °C until assay.

Of the 55 participants who provided saliva samples, 19 could not be assayed owing to a loss of samples during a laboratory personnel transition. This loss was unrelated to participant characteristics, as samples were lost as a batch rather than selectively, and the missingness is therefore considered to be completely at random with respect to the study variables.

Awakening times were based on participant self-report. Objective verification of awakening time using actigraphy or electronic cap-monitoring devices (e.g., TrackCaps) was not employed, which is acknowledged as a limitation of the cortisol protocol.

#### 2.3.3. Cortisol Assay Procedure

Salivary cortisol was quantified using liquid chromatography tandem mass spectrometry (LC-MS/MS) at Merrimack College’s Shared Instrumentation and Research Facility (SIRF) on an Agilent 6420 triple quadrupole system (Agilent Technologies, Santa Clara, CA, USA) equipped with an electrospray ionization source, following a method adapted from Bakusic et al. (2019). Prior to analysis, samples underwent solid-phase extraction using Oasis Prime HLB cartridges (Waters Corporation, Milford, MA, USA). Chromatographic separation was performed on an Agilent Eclipse Plus C18 column (1.8 µm, 2.1 × 50 mm; Agilent Technologies, Santa Clara, CA, USA) using a gradient mobile phase of 0.1% formic acid in water and 0.1% formic acid in methanol. Cortisol was monitored using a quantifier transition of 363.3 → 120.6 (m/z) and a qualifier transition of 363.3 → 90.5 (m/z). A stable isotope-labeled internal standard (cortisol-d4; Thermo Fisher Scientific, Waltham, MA, USA) was added to each sample prior to extraction to correct for extraction variability and ion suppression. Cortisol-d4 was monitored at 120.7 (m/z). Calibration curves were constructed from serial dilutions ranging from 0.1 ng/mL to 50 ng/mL, spiked with 20 ng/mL cortisol-d4, and fit using weighted (1/x^2^) linear regression on the ratios of cortisol to internal standard peak areas. All samples from a given participant were analyzed within the same analytical batch to minimize between-run variability.

The cortisol awakening response was operationalized as the area under the curve with respect to increase (AUCi), calculated from the four morning samples using the trapezoid formula (Pruessner et al., 2003). AUCi specifically captures the rise in cortisol from awakening across the 45-min window and is the recommended index for CAR research (Pruessner et al., 2003; Stalder et al., 2016). Cortisol samples were screened for protocol noncompliance and extreme values. No samples exceeded ±3 standard deviations from the sample mean, and therefore no observations were excluded on this basis. Participants who failed to provide at least three of the four samples were excluded from CAR analyses.

#### 2.3.4. Risk-Taking Behavioral Task

Following saliva sample collection, participants came into the lab to complete the Balloon Emotional Learning Task (BELT; Humphreys et al., 2013), a behavioral task assessing risk-taking and implicit learning under uncertainty. The BELT is a computerized task in which participants inflate virtual balloons to earn points, with each pump increasing potential reward but also the risk of the balloon exploding (i.e., “popping”), resulting in the loss of points for that trial. The task includes three conditions that vary in predictability and reward structure. Two conditions are stable, with fixed explosion thresholds corresponding to either relatively low (e.g., 6 pumps) or high (e.g., 18 pumps) allowable pump limits. In the variable (uncertain) condition, the explosion point varies across trials (e.g., 6, 12, or 18 pumps), requiring participants to balance reward maximization against uncertainty-driven risk.

Participants completed a total of 54 trials, with equal representation of each condition (18 trials per condition). Participants were informed that balloons of different colors represented materials that differed in strength, with some more likely to withstand greater expansion and others more prone to popping. However, they were not informed which colors corresponded to each condition. They were only informed that some balloons were stronger than others and could therefore withstand more pumping before they popped. Thus, participants were required to implicitly learn which balloon types yielded greater reward value. Primary behavioral outcomes included total points earned (indexing overall task performance), total pumps in the variable condition (indexing risk-taking under uncertainty), and total number of pops (indexing maladaptive risk-taking).

The BELT was selected in preference to the standard Balloon Analogue Risk Task because its condition structure, comprising two stable explosion thresholds and one variable threshold that are not identified to participants, permits risk-taking under uncertainty to be distinguished from risk-taking under predictable reward contingencies (Humphreys et al., 2013, 2016). This distinction was central to our second hypothesis, which concerned risk-taking under uncertainty specifically.

#### 2.3.5. Questionnaires

All participants were asked to complete a set of questionnaires via REDCap (Research Electronic Data Capture, version 14.1.6), a secure, web-based software platform designed to support data capture for research studies (P. A. Harris et al., 2009; P. A. Harris et al., 2019). Questionnaires included health and demographics, as well as standardized questionnaires about sleep behaviors and personality. Particularly, participants completed the Pittsburgh Sleep Quality Index (PSQI; Buysse et al., 1989), which includes 19 items assessing sleep quality over the past month and reports on several measures of subjective sleep quality, including sleep duration. The Morningness-Eveningness Questionnaire (MEQ; Horne & Ostberg, 1976) assesses chronotype across 19 items on a 4–6-point scale, with higher scores indicating greater morningness. The Behavioral Inhibition Scale/Behavioral Activation Scales (BIS/BAS; Carver & White, 1994), comprise 24 items rated on a 4-point scale, yielding a BIS score indexing sensitivity to punishment and three BAS subscales (Drive, Fun Seeking, Reward Responsiveness) indexing sensitivity to reward, and the UPPS Impulsive Behavior Scale (Whiteside & Lynam, 2001) assesses four dimensions of impulsivity (urgency, lack of premeditation, lack of perseverance, sensation seeking) across 20 items rated on a 4-point scale.

### 2.4. Statistical Analyses

All analyses were conducted in R (version 2024.12.1). Linear regression models were used throughout unless otherwise noted. Sleep analyses were restricted to participants who obtained at least 3 h of sleep during the recording night in order to ensure reliable estimates of sleep physiology. Primary analyses were hypothesis-driven and focused on a limited set of behavioral outcomes derived from the Balloon Emotional Learning Task (BELT). Based on a priori predictions, two primary models were specified. First, a linear regression model tested whether rapid eye movement (REM) sleep percentage predicted overall task performance, indexed by total points earned. Second, linear regression models tested whether N2-N3 sigma power predicted indices of risk-taking behavior, including total pumps in the variable (uncertain) condition and total number of pops. All models included age as a covariate.

Assumptions of linear regression were evaluated for all primary regression models. Residual and Q–Q plots were visually inspected to assess linearity and normality of residuals. Homoscedasticity was evaluated using residual-versus-fitted plots and Breusch–Pagan tests. Influential observations were assessed using Cook’s distance and leverage statistics, and variance inflation factors (VIFs) were examined to evaluate multicollinearity. No violations were identified that were judged to meaningfully affect interpretation of the primary analyses.

Cortisol analyses were conducted in a subsample of participants who provided at least three of the four morning saliva samples and also had complete BELT data (*n* = 28). Because AUCi values were positively skewed, they were log-transformed prior to all analyses to meet the assumptions of linear regression. To examine whether the CAR was associated with risk-taking behavior, separate linear regression models tested whether log-transformed AUCi predicted each of the three primary behavioral outcomes: total points earned, total pumps in the variable condition, and total number of pops. Age was included as a covariate in all models. To test whether the CAR moderated the relationship between sleep physiology and behavioral performance, a moderation model was specified with REM sleep percentage and log-transformed AUCi as predictors of total points earned, including their interaction term (REM × AUCi). Finally, to examine whether the CAR was associated with objective sleep physiology, separate linear regression models tested associations between log-transformed AUCi and both REM time and N2-N3 sigma as primary measures of interest. Exploratory analyses also examined associations between AUCi and sleep duration, sleep efficiency, wake after sleep onset, N2-N3 delta activity, NREM2 time, and NREM3 time. Datasets analyzed for this study have been made available in the Open Science Framework repository.

Sensitivity analyses were conducted to determine the minimum effect sizes detectable at 80% power and α = 0.05. For the sleep analyses (*n* = 50), the study was powered to detect bivariate correlations of approximately r = 0.39 or larger, and effects of f^2^ = 0.16 (partial R^2^ = 0.14) for a focal predictor in models including age as a covariate. For the cortisol analyses, sensitivity analyses were conducted using the final analytic sample sizes. For the regression examining log-transformed cortisol AUCi as a predictor of total points earned while controlling for age (*n* = 28), the minimum detectable effect at α = 0.05 with 80% power was Cohen’s f^2^ = 0.315 (partial R^2^ = 0.239), approaching the conventional threshold for a large effect (f^2^ = 0.35). For the moderation analysis examining the REM percentage × cortisol AUCi interaction while controlling for the corresponding main effects and age (*n* = 27), the minimum detectable interaction effect was f^2^ = 0.358 (partial R^2^ = 0.264), exceeding the conventional large-effect threshold. These analyses were therefore underpowered to detect small to medium effects, and null findings from the cortisol and moderation analyses should be interpreted with caution given the elevated risk of Type II error.

Exploratory analyses examined whether self-reported chronotype, personality, and impulsivity were associated with sleep physiology, cortisol awakening response (CAR), and behavioral performance. Separate linear regression models were conducted to examine associations between Morningness–Eveningness Questionnaire (MEQ) scores and sleep physiology (REM sleep percentage, total sleep time, sleep efficiency) and log-transformed AUCi, while controlling for age. Additional linear regression models examined associations between Behavioral Inhibition System/Behavioral Activation System (BIS/BAS) and UPPS-P impulsivity subscale scores with BELT behavioral outcomes and log-transformed AUCi, also controlling for age. Because these analyses involved multiple exploratory comparisons, Benjamini–Hochberg false discovery rate (FDR) correction was applied across the family of sleep–personality analyses to control the expected false discovery rate. Finally, to evaluate potential bias due to missing questionnaire data, participants who completed the questionnaire battery were compared with non-completers on demographic, sleep, cortisol, and behavioral measures using Welch’s independent-samples *t*-tests. Group differences were quantified using Hedges’ *g* to estimate effect sizes.

## 3. Results

### 3.1. Descriptive Statistics

Descriptive statistics for sleep, behavioral, and hormonal variables are presented in Table 1. On average, participants slept for 5.81 h (min-max = 3.175–8.767 h; *SD* = 1.28) with a mean sleep efficiency of 84.05% (*SD* = 8.75). Sleep duration did not differ substantially by developmental stage: participants aged 15–17 slept an average of 5.58 h (*SD* = 1.23), and participants aged 18 and older slept an average of 6.12 h (*SD* = 1.24), *t*(41.18) = −1.50, *p* = 0.141, 95% CI [−1.28, 0.19].

To evaluate whether the relatively short total sleep time observed during the recording night reflected an artifact of the recording device, objective total sleep time recorded by the Sleep Profiler was compared with participants’ self-reported habitual sleep duration from the PSQI. Objective and subjective sleep duration were strongly correlated (*r* = 0.67, *p* < 0.001), indicating that participants who reported longer habitual sleep also slept longer during the recording night. Although objective total sleep time was, on average, 0.84 h shorter than habitual self-reported sleep duration (*t*(37) = −5.41, *p* < 0.001), this difference is consistent with expected night-to-night variability and first-night effects associated with objective sleep monitoring. Mean global PSQI score was 6.14 (*SD* = 2.09), just above the established clinical threshold for poor sleep quality (≥5), consistent with the elevated rates of sleep disruption characteristics of this population.

### 3.2. Primary Analyses

**Figure 2:**
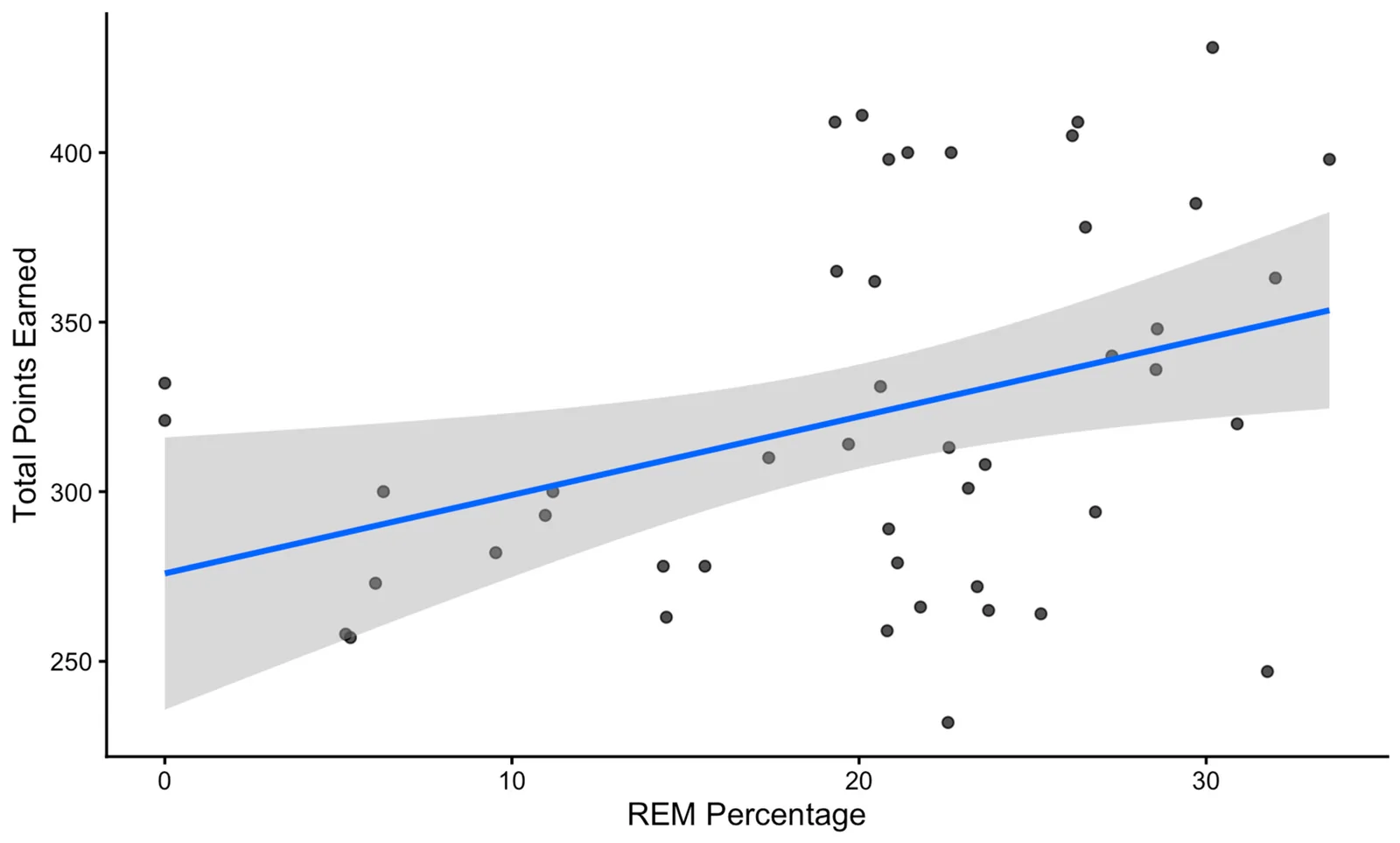
Association between REM percentage and total points earned on the BELT. Higher REM percentage was associated with greater overall performance. Each point represents an individual participant. The blue line represents the fitted linear regression line and the shaded area indicates 95% confidence interval.

Analyses were restricted to participants who obtained at least 3 h of sleep (*n* = 46). Age was included as a covariate in all models. Primary hypotheses tested associations between REM sleep and N2-N3 sigma activity with overall task performance and indices of risk-taking behavior. Consistent with predictions, REM percentage was positively associated with overall performance on the Balloon Emotional Learning Task (BELT), such that higher REM sleep was associated with greater total points earned, (*b* = 18.60, β = 0.34, *SE* = 7.50, *t* = 2.48, *p* = 0.017, 95% CI [3.49, 33.70], *R*^2^ = 0.181, adjusted *R*^2^ = 0.143; Figure 2).

**Figure 3:**
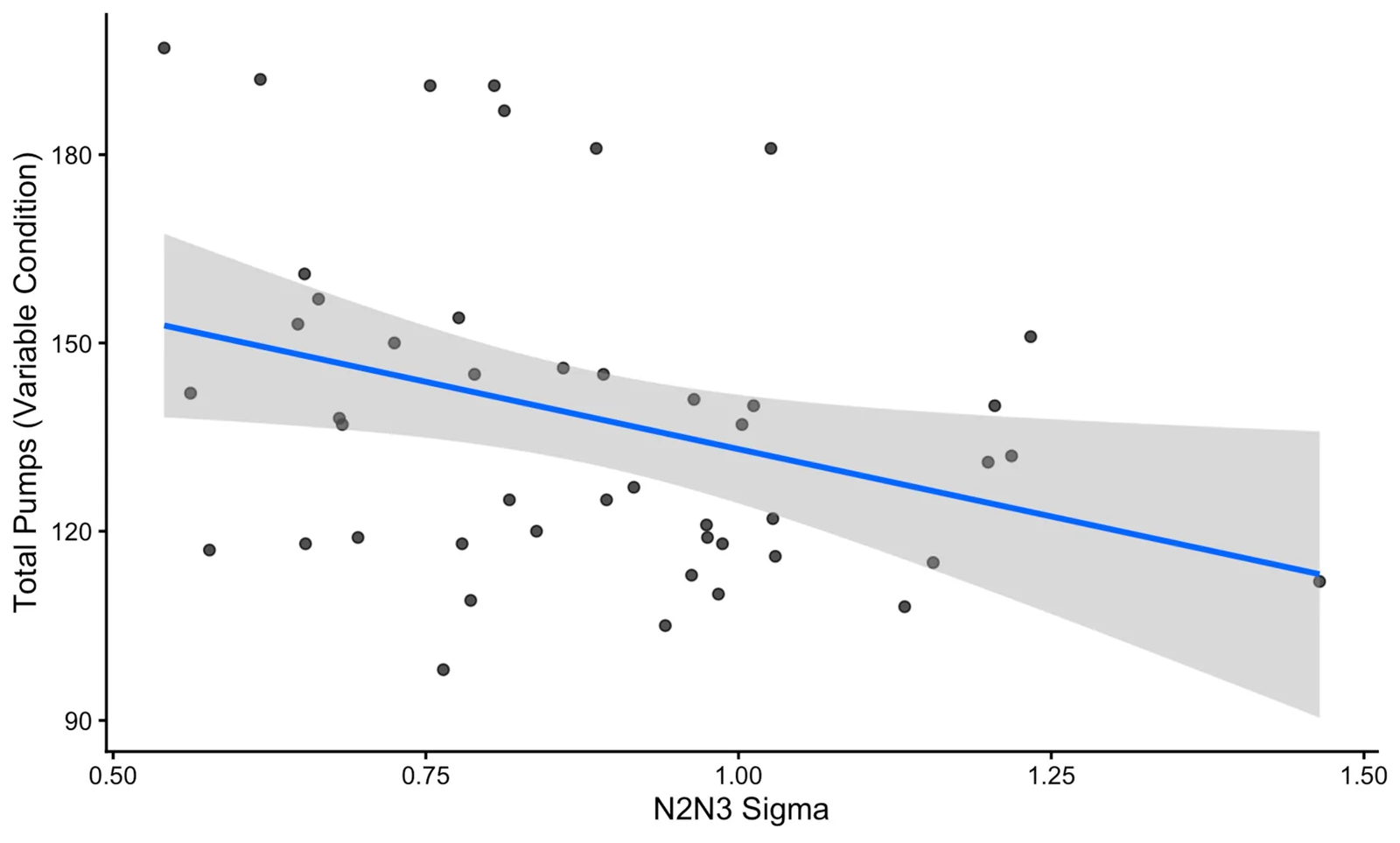
Association between N2-N3 sigma band spectral power (12–16 Hz) and total pumps in the variable condition. Higher sigma power was associated with reduced risk-taking under uncertainty. Each point represents an individual participant. The blue line represents the fitted linear regression line and the shaded area indicates 95% confidence interval.

**Figure 4:**
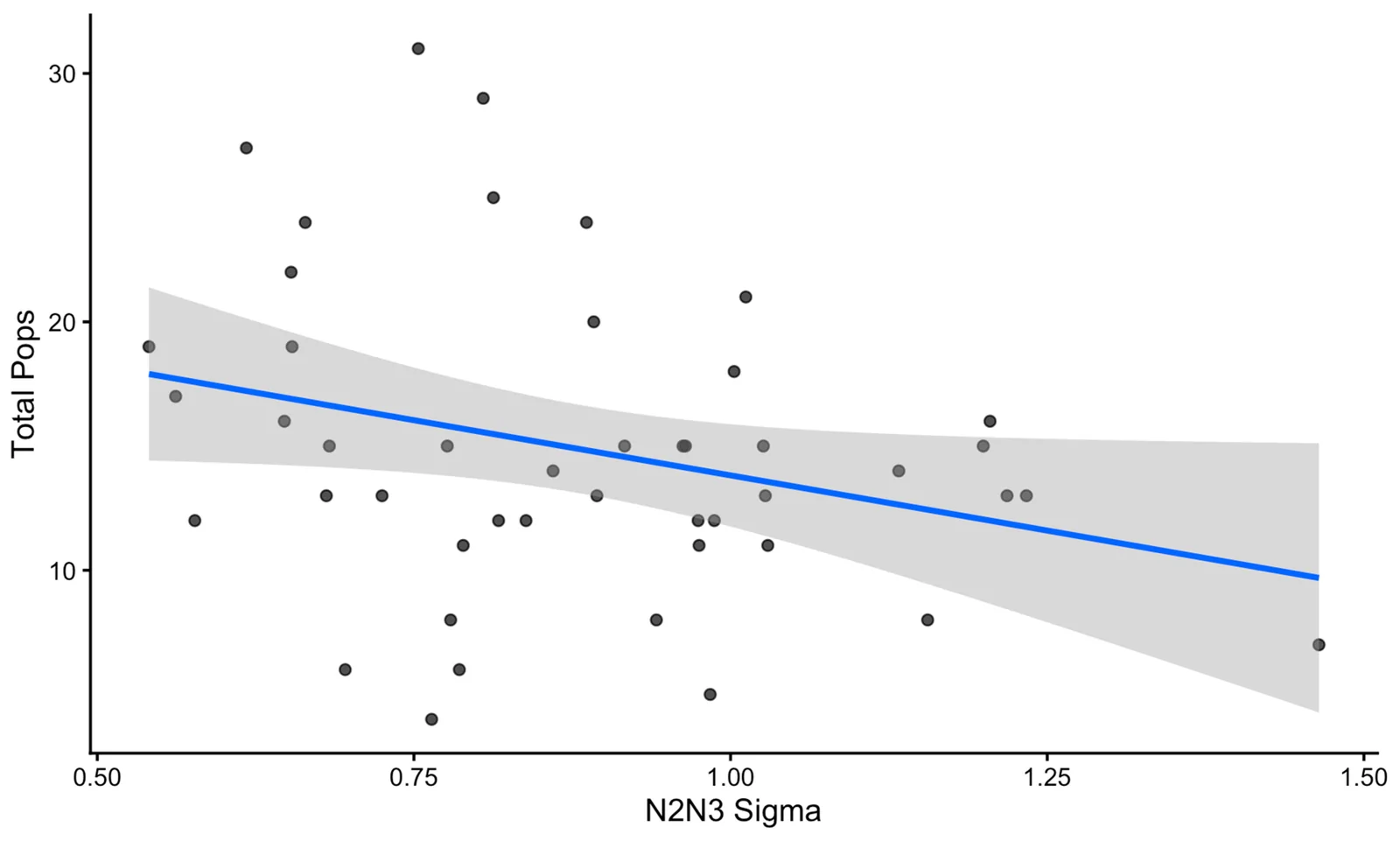
Association between N2-N3 sigma band spectral power (12–16 Hz) and total pops on the BELT. Higher sigma power was associated with fewer pops, indicating reduced maladaptive risk-taking. Each point represents an individual participant. The blue line represents the fitted linear regression line and the shaded area indicates 95% confidence interval.

N2-N3 sigma activity was associated with reduced risk-taking behavior. Specifically, higher sigma power predicted fewer pumps in the variable (uncertain) condition (*b* = −9.77, β = −0.37, *SE* = 4.09, *t* = −2.39, *p* = 0.021, 95% CI [−18.00, −1.53], *R*^2^ = 0.122, adjusted *R*^2^ = 0.081; Figure 3). Additionally, higher sigma power predicted fewer total pops (*b* = −2.07, β = −0.34, *SE* = 0.96, *t* = −2.15, *p* = 0.037, 95% CI [−4.02, −0.13], *R*^2^ = 0.115, adjusted *R*^2^ = 0.074; Figure 4).

As an exploratory analysis, we examined whether subjective sleep quality, measured using the Pittsburgh Sleep Quality Index (PSQI), predicted BELT performance while controlling for age. PSQI scores were not associated with total points earned (*b* = 2.43, *SE* = 4.13, *p* = 0.560), total pops (*b* = −0.32, *SE* = 0.42, *p* = 0.443), or adjusted pumps in the variable condition (*b* = −0.13, *SE* = 0.14, *p* = 0.377). This suggests that specific aspects of sleep physiology may be more sensitive predictors of emotional risk-taking than global self-reported sleep quality.

Because the study focused on adolescents and emerging adults, sensitivity analyses were conducted excluding one participant who was 34 years old, approximately 10 years older than the next oldest participant (24 years). Excluding this participant did not meaningfully alter the primary findings. REM sleep remained positively associated with total points earned (*b* = 2.28, *SE* = 0.90, *p* = 0.015), and N2-N3 sigma activity remained negatively associated with adjusted pumps in the variable condition (*b* = −3.25, *SE* = 1.38, *p* = 0.023), while the association with total pops remained at trend level (*b* = −8.73, *SE* = 4.41, *p* = 0.054). Furthermore, interaction analyses indicated that the associations between sleep measures and BELT performance did not differ significantly between adolescents and young adults (all interaction *ps* ≥ 0.335).

### 3.3. Cortisol Analyses

**Figure 5:**
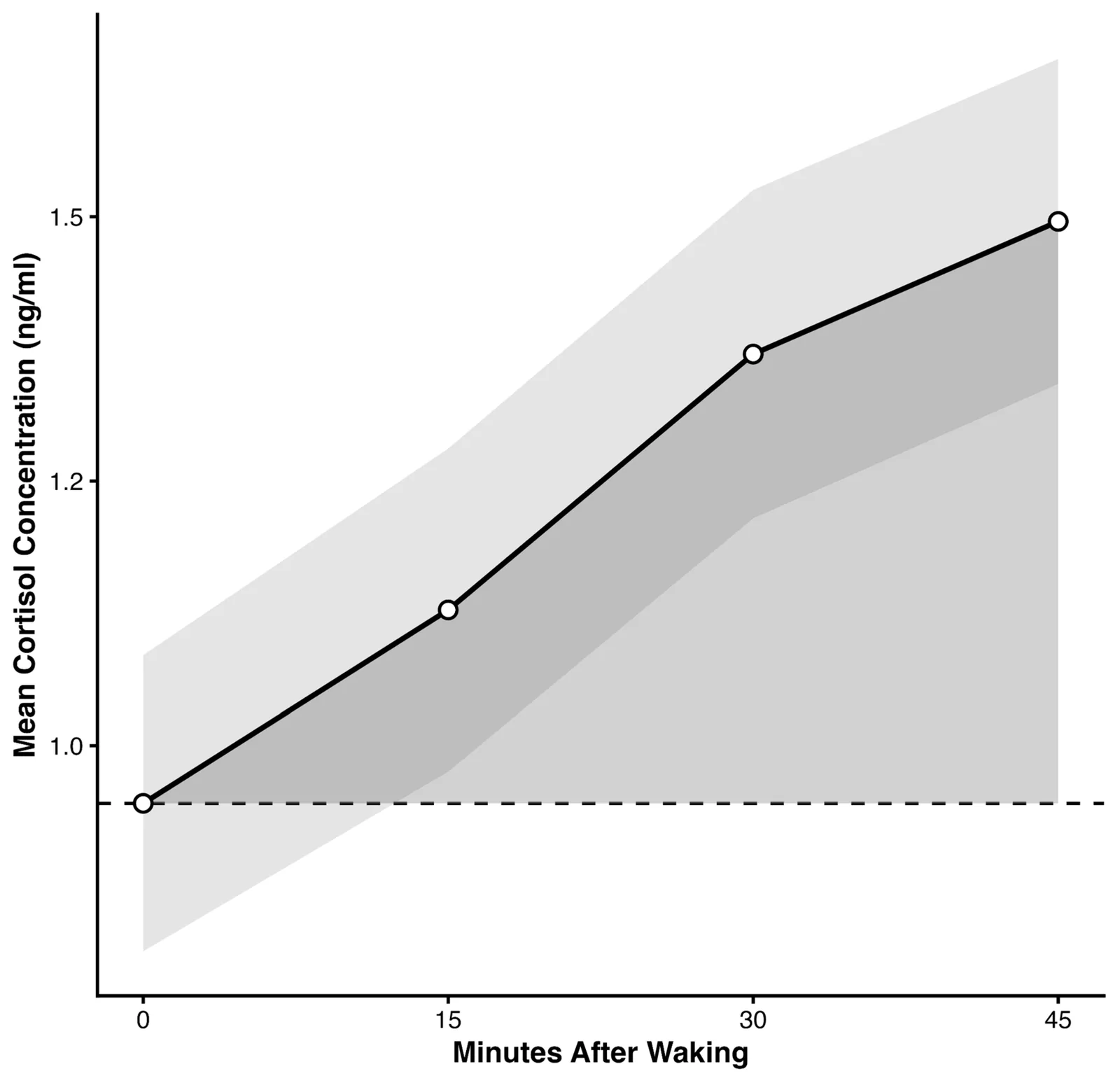
Mean cortisol awakening response (AUCi) across the first 45 min following awakening. Cortisol concentrations were measured immediately upon awakening and again at 15-, 30-, and 45-min intervals. Shaded region under the fit line represents the area under the curve with respect to increase (AUCi), indexing dynamic cortisol responsivity relative to the awakening baseline. Shaded error band around the fit line represents 95% confidence intervals around the mean.

**Figure 6:**
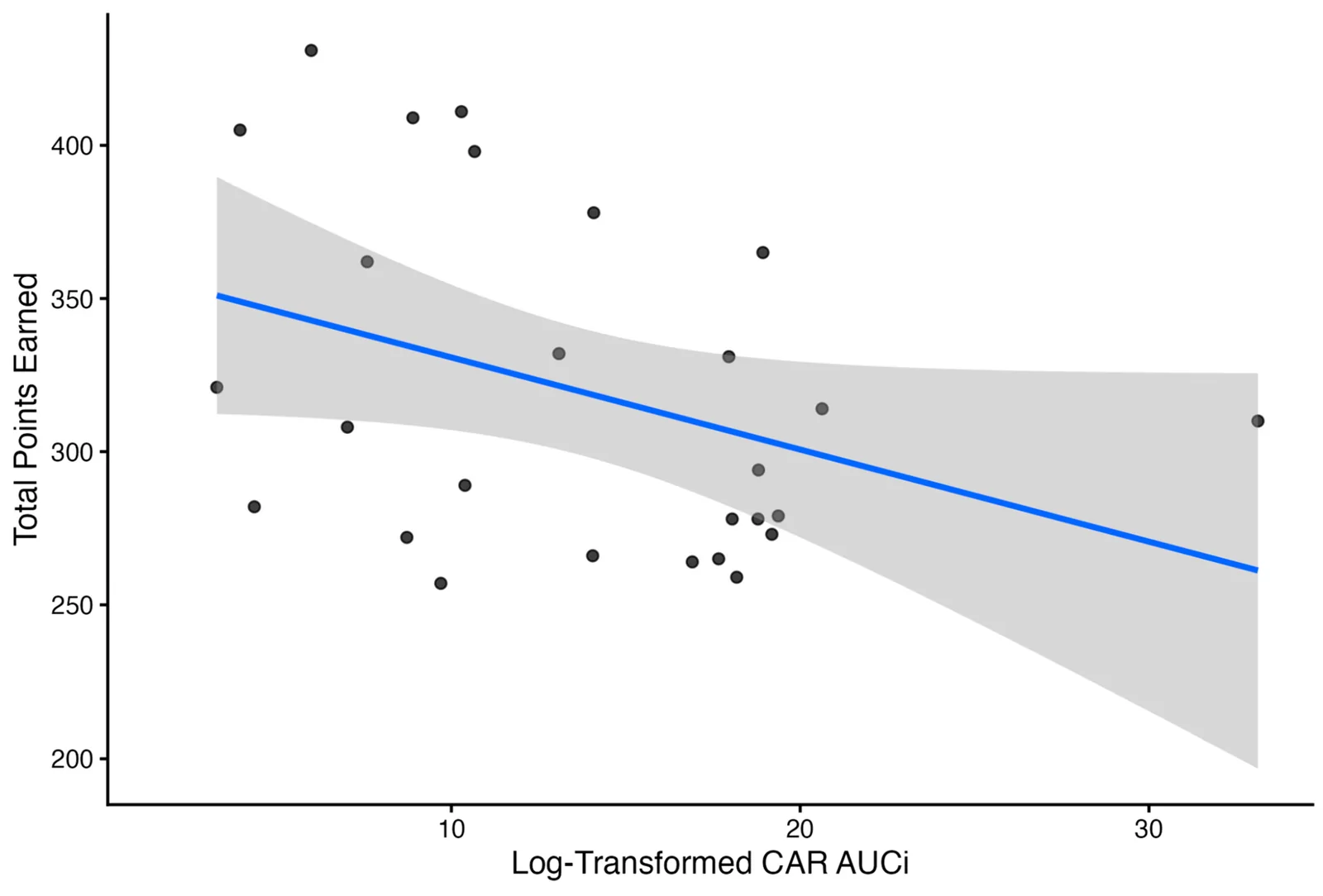
Association between log-transformed cortisol awakening response (AUCi) and total points earned on the BELT. Greater cortisol responsivity following awakening was associated with fewer total points earned, suggesting that heightened endocrine stress sensitivity may relate to more cautious or less reward-driven behavioral responding. Each point represents an individual participant. The blue line represents the fitted linear regression line and the shaded area indicates 95% confidence interval.

A total of 28 participants provided complete cortisol awakening response (CAR) data and were included in hormonal analyses (Figure 5). Because cortisol values were positively skewed, AUCi values were log-transformed prior to analysis. Within the cortisol subsample, log-transformed AUCi demonstrated a significant main effect on total points earned, such that greater CAR responsiveness was associated with fewer points earned on the task (*b* = −25.90, β = −0.41, *SE* = 10.90, *p* = 0.026; Figure 6). Neither REM percentage in this smaller subset nor the REM × AUCi interaction significantly predicted total points earned (*ps* > 0.16), indicating that CAR was independently related to behavioral performance but did not moderate associations between REM sleep and risk-taking behavior. AUCi was also not significantly associated with total pops or total pumps in the variable condition (*ps* > 0.15). Log-transformed AUCi was not significantly associated with objective sleep measures, including sleep duration, sleep efficiency, wake after sleep onset, N2-N3 delta activity, N2-N3 sigma activity, REM time, NREM2 time, or NREM3 time (all *ps* > 0.33). Together, these findings suggest that sleep physiology and endocrine responsivity may contribute independently to behavioral responding, rather than through an interactive moderation process, within this sample.

### 3.4. Questionnaire Analyses

Previous work has shown that chronotype may influence sleep behavior (Horváth et al., 2025; Mongrain et al., 2005; Vitale et al., 2015), as well as morning cortisol, with some studies reporting higher morning cortisol in morning types (Kudielka et al., 2006; Randler & Schaal, 2010). Findings for the CAR specifically are mixed, however, and studies employing AUCi report no association (e.g., Griefahn & Robens, 2008; Petrowski et al., 2020). We therefore wanted to assess whether participant morning/evening preferences played a role in our study. The relationship between chronotype and sleep physiology was examined using raw Morningness–Eveningness Questionnaire (MEQ) scores while controlling for age. Greater morning preference was associated with a higher percentage of REM sleep (*b* = 0.41, *SE* = 0.20, *p* = 0.047, 95% CI [0.01, 0.81]). In contrast, MEQ scores were not significantly associated with log-transformed cortisol awakening response (AUCi), consistent with previous studies (Griefahn & Robens, 2008; Petrowski et al., 2020), total sleep time, or sleep efficiency (all *ps* > 0.30). Age was not significantly associated with any outcome measure (all *ps* > 0.23).

Additionally, we wanted to examine whether individual trait differences in personality impacted the relationships observed in this study. The BIS/BAS was used as a measure of sensitivity to punishment (BIS) and to reward (BAS), which are highly relevant to performance on the BELT. In addition, the UPPS was used as a measure of impulsivity, which may independently contribute to risk-taking behavior on the BELT. When controlling for age, higher Behavioral Inhibition System (BIS) scores were associated with fewer total balloon pops (*b* = −0.53, *SE* = 0.23, *p* = 0.030), indicating that individuals with greater sensitivity to potential negative outcomes exhibited less risky behavior. A trend-level negative association was also observed between BIS scores and total pumps in the variable condition (*b* = −0.15, *SE* = 0.08, *p* = 0.081). However, these associations did not remain statistically significant following Benjamini–Hochberg false discovery rate correction for multiple comparisons. No significant associations were observed for Behavioral Activation System (BAS) subscales or UPPS impulsivity dimensions (all *ps* > 0.19).

As a secondary analysis, we examined whether trait behavioral inhibition moderated the associations between sleep physiology and BELT performance. Separate regression models tested BIS × sleep interaction terms for the primary associations identified in the study (REM sleep × BIS predicting total points earned; N2-N3 sigma × BIS predicting total pops and adjusted pumps in the variable condition). No significant interaction effects were observed (all *ps* ≥ 0.64; all Benjamini–Hochberg FDR-adjusted *ps* ≥ 0.69), indicating that trait behavioral inhibition did not significantly modify the relationships between sleep physiology and risk-taking behavior.

Associations between cortisol awakening response (log-transformed AUCi) and personality measures were examined while controlling for age. No significant associations were observed between CAR and BIS, BAS Drive, BAS Fun Seeking, BAS Reward Responsiveness, or UPPS impulsivity dimensions (all *ps* > 0.15), and these findings remained unchanged following Benjamini–Hochberg correction for multiple comparisons. These findings suggest that individual differences in endocrine stress responsivity were not strongly related to trait measures of behavioral inhibition, reward sensitivity, or impulsivity in this sample.

To evaluate potential bias resulting from missing questionnaire data, participants who completed the questionnaire battery (*n* = 39–38, depending on variable) were compared with non-completers (*n* = 10–14). Groups did not differ significantly with respect to age, total sleep time, REM sleep percentage, N2-N3 sigma activity, or log-transformed cortisol awakening response (all FDR-adjusted *ps* ≥ 0.34). Effect sizes for these comparisons were small (Hedges’ *g*s = 0.16–0.41), suggesting that missing questionnaire data were unlikely to systematically bias the questionnaire analyses.

## 4. Discussion

The present study examined associations among objective sleep architecture, the cortisol awakening response (CAR), and risk-taking behavior in a sample of adolescents and young adults. Consistent with predictions, both REM sleep percentage and N2-N3 sigma activity were associated with next-morning behavioral performance on the BELT, with each sleep variable predicting distinct behavioral outcomes. The CAR was independently associated with overall task performance, but contrary to our predictions, CAR did not moderate the relationship between sleep and behavioral associations, nor was it significantly associated with objective sleep measures. Together, these findings support the notion that specific features of sleep architecture may have stage-specific implications for risk-taking behavior, and that sleep physiology and morning cortisol responsivity may operate as distinct physiological contributors to behavioral outcomes.

### 4.1. Interpretation of Primary Findings

Higher REM sleep percentage was associated with greater overall performance on the BELT, consistent with the established role of REM sleep in the consolidation of emotional memories (Nishida et al., 2009; Wagner et al., 2001), though the specific contributions of REM versus slow wave sleep to this process remain an active area of investigation (Rawson & Jackson, 2024). This is also consistent with the role of REM sleep in the regulation of prefrontal-limbic circuitry (Hong et al., 2023; Muzur et al., 2002). REM sleep is characterized by heightened activity in the insula, medial prefrontal cortex, and amygdala (see review: Goldstein & Walker, 2014). These brain regions are central to the salience neural network and to the evaluation of reward and risk (Ben Shalom, 2022). The present finding extends this literature by demonstrating that naturally occurring individual differences in REM sleep percentage are associated with behavioral performance on a task requiring implicit learning and reward maximization (Humphreys et al., 2013, 2016) in a community sample of adolescents and young adults. This is particularly notable given that this population is already at elevated risk for REM sleep disruption due to circadian phase delay and environmental pressures on sleep timing.

Higher N2-N3 sigma power predicted both fewer pumps in the variable condition and fewer total pops, suggesting that NREM sigma-band activity is associated with more conservative, adaptive risk-taking behavior under uncertainty. Sigma-band power reflects integrated spectral energy in the 12–16 Hz range, a frequency band dominated by sleep spindle activity (e.g.,: Fogel & Smith, 2011; Campbell & Feinberg, 2016), and one plausible interpretation of this association draws on the proposed role of spindles in strengthening hippocampal–prefrontal connectivity overnight (Andrade et al., 2011; Cowan et al., 2020), a consolidation process that may support top-down regulation of impulsive responding the following morning (Fang et al., 2019; Mark Lawrence et al., 2020). We emphasize that our measure does not permit attribution of this effect to spindle activity specifically, or to a particular spindle parameter. The specificity of this finding to the variable condition, when explosion thresholds were unpredictable, is noteworthy as it suggests that the processes indexed by sigma activity may be particularly relevant for navigating uncertainty the next day, rather than for performance under stable, predictable conditions.

Greater CAR was associated with poorer overall task performance, suggesting that heightened adrenal sensitivity at awakening may relate to more cautious or less reward-driven behavioral responding. While much of the literature suggests that elevated cortisol is associated with greater risk-taking and reward-seeking behavior, this relationship is modified by a number of factors (Duque et al., 2022), including sex: cortisol has been found to increase risky decision-making in men but not in women, and may reduce risk-taking in women, potentially reflecting decreased reward responsiveness under stress (Kluen et al., 2017). Given that our sample was predominantly female, the more conservative responding observed here is consistent with this pattern. There is also evidence that consistent cortisol elevation can shift behavioral responding toward more conservative choices under uncertainty (Kandasamy et al., 2014). Critically for this study, the CAR does not reflect situational stress at the time of testing (Stalder et al., 2025). Rather, the CAR reflects adrenal sensitivity to adrenocorticotropic hormone (ACTH; Abelson et al., 2023; Schmidt-Reinwald et al., 1999), suggesting that what we captured in this study is stable individual differences in HPA axis reactivity rather than an index of acute stress. Higher adrenal sensitivity may therefore prime individuals toward a particular physiological state at the start of the day. This is consistent with proactive accounts of the CAR, in which morning cortisol configures neural systems in advance of anticipated demands, with domain-specific rather than uniform effects (Xiong et al., 2021). We note, however, that the direction of the association observed here differs from that reported in this work, which may again reflect the sample composition noted above. One such domain-specific effect is that higher CAR has been associated with reduced neural engagement with negative stimuli (Shi et al., 2022), raising the possibility that individuals with greater adrenal sensitivity may be less attuned to the performance-relevant negative outcomes, such as balloon pops and point losses, that are necessary for optimal implicit learning on the BELT. This interpretation remains preliminary; however, as Shi et al. (2022) examined responses to emotionally unpleasant images rather than performance-based feedback, future work should directly examine whether CAR modulates neural responses to negative feedback in decision-making contexts.

Notably, the CAR did not moderate the relationship between REM sleep and task performance, nor was it significantly associated with objective sleep measures, suggesting that sleep physiology and morning cortisol responsivity may represent dissociable pathways. This apparent independence is consistent with a mixed literature: associations between sleep duration, sleep quality and the CAR vary in magnitude and direction across studies (Elder et al., 2014), and evidence bearing specifically on sleep architecture is sparser still, with at least one study using controlled circadian protocols reporting no association between REM or NREM proportion and the CAR (Bowles et al., 2022). While the HPA axis is regulated in part by sleep stage activity (Backhaus et al., 2004; Devine & Wolf, 2016), the present findings suggest that individual differences in cortisol responsivity at awakening are not straightforwardly predicted by the sleep architecture features examined here. One possible explanation for this independence may be related to the distinction between the two regulatory processes governing sleep and waking physiology. The CAR may more closely reflect circadian regulation (Process C), which operates on a stable, slowly shifting timescale, rather than homeostatic sleep pressure (Process S), which is more directly sensitive to the quality and architecture of a given night’s sleep (Borbély, 1982). Consistent with this, the CAR has been shown to be modulated by the circadian system independent of sleep behavior (Bowles et al., 2022). This would explain why individual differences in REM sleep or sigma activity, both reflective of Process S dynamics, did not significantly predict cortisol responsivity at waking. However, it is important to note that the CAR appears to require the awakening trigger (Vargas & Lopez-Duran, 2020; Wilhelm et al., 2007), meaning that it is also not purely driven by circadian factors. It should also be noted that the CAR has been shown to exhibit moderate trait-like stability across aggregated assessments within individuals (Wüst et al., 2000a), suggesting that it may reflect relatively stable differences in HPA axis reactivity that are not easily perturbed by a single night’s variation in sleep architecture. These interpretations should also be treated as provisional. Cortisol data were available for 36 participants (*n* = 27/28 included in analyses), and the analyses were correspondingly underpowered; the absence of a significant association should not be read as evidence that no relationship exists. Distinguishing genuine independence of these systems from insufficient sensitivity to detect a modest association will require larger samples and repeated sampling across nights. Additionally, cortisol was assessed on a single morning. While this design is appropriate for testing whether a given night’s sleep relates to the following morning’s cortisol response, a single paired observation per participant cannot distinguish night-to-morning coupling from stable individual differences in both measures, and is subject to any measurement error arising from variability in sampling adherence. Repeated assessment across multiple nights would permit these to be separated in future studies.

### 4.2. Methodological Limitations and Interpretive Considerations

Several limitations of the present study should be acknowledged. The present design is cross-sectional and correlational, and cannot establish causal direction. Although we have framed these findings in terms of sleep-dependent processes influencing next-morning behavior, the reverse is equally consistent with the data, and a stable individual difference could plausibly underlie both. Trait impulsivity (UPPS) and reward sensitivity (BAS) were not significantly associated with behavioral outcomes or the CAR, which argues against these particular traits as common causes; these analyses were themselves underpowered, however, and therefore the possibility of traits confounding the results cannot be excluded. Establishing directionality will require longitudinal or experimental designs.

Additionally, the overall sample size was modest and predominantly female (86%), which limits the generalizability of the findings and prevented us from examining gender as a moderator of the associations reported here. This is particularly relevant to the interpretation of our cortisol findings, as the association between cortisol and risk-taking appears to be sex-dependent (Kluen et al., 2017); the more conservative responding observed here is therefore consistent with our sample composition, but this account requires testing in samples with greater sex balance. Further, we did not assess the menstrual phase while individuals participated in the study. Sex differences and menstrual phase have been shown to impact sleep behavior (Cherukuri et al., 2026; Driver & Baker, 1998), and risky decision-making (Charness & Gneezy, 2012; C. R. Harris & Jenkins, 2006). However, sex differences and menstrual phase have been shown not to impact the CAR in adolescents (Bouma et al., 2009). Hormonal contraceptive use, by contrast, has been associated with a blunted CAR (Bouma et al., 2009). As contraceptive use was not recorded, we cannot determine what proportion of our sample was affected, nor adjust for it; unmeasured variation in contraceptive use may therefore have contributed to variability in our cortisol measures. Future research should replicate these findings in samples with greater sex balance and examine whether the associations between sleep architecture, cortisol responsivity, and risk-taking behavior differ as a function of sex—questions the present study was not designed or powered to address.

Participants were recruited both from the local community and through a university participant pool, and the sample was demographically comparable to national population estimates with respect to racial identity, with Hispanic and Latinx participants somewhat over-represented. Recruitment was nonetheless restricted to a single geographic region. Given that both sleep duration and risk-taking behavior vary with residential environment, the present findings may not generalize to young people in materially different contexts, and we are unable to evaluate the role of socioeconomic factors in the associations reported here.

Substance use, including caffeine, nicotine, and alcohol, was neither an exclusion criterion nor assessed, despite known effects on both sleep architecture and cortisol secretion. Reporting bias may also differ by age group, as participants under 18 participated with parental consent.

Further, cortisol analyses were conducted in a reduced analytic sample (*n* = 27–28, depending on the model), reducing statistical power for cortisol analyses. Additionally, we did not have a measure of the circadian phase in which the participants were awakening, nor did we require that they wake naturally rather than use an alarm. Furthermore, awakening times were based on participant self-report without objective verification via actigraphy or electronic compliance monitoring devices, which may have introduced variability in the timing of saliva samples relative to true awakening. This could have impacted our measurement of the CAR, as waking too early relative to their circadian phase may have blunted the AUCi, although research suggests that these factors may also have no influence over the CAR (Stalder et al., 2009; Wüst et al., 2000b).

Another limitation of the study was that sleep was assessed using the Sleep Profiler, a frontopolar EEG device rather than conventional polysomnography. Although this approach does not include occipital EEG derivations or dedicated EOG channels, validation studies have demonstrated sleep-stage agreement comparable to inter-scorer reliability for N2, N3, and REM sleep (Levendowski et al., 2017, 2023). While agreement is lower for N1 sleep, the primary outcomes in the present study were REM sleep percentage and N2-N3 sigma activity, sleep metrics for which the Sleep Profiler has demonstrated good agreement with conventional polysomnography. Consequently, this limitation is unlikely to have substantially affected the interpretation of the present findings. Lastly, sigma power is a less sensitive index of discrete spindle events than automated event detection, as spindles occupy only a small proportion of the NREM interval over which power is computed (Palepu et al., 2023). The associations we report were nonetheless detectable using this coarser index, though they require replication with event-level measures before conclusions can be drawn at the level of specific spindle parameters.

REM sleep percentage and N2-N3 sigma activity were selected a priori on mechanistic grounds, and other recorded dimensions of sleep, including sleep efficiency and wake after sleep onset, were not examined as predictors of task performance. As sleep is multidimensional and continuity measures may relate to next-day functioning through distinct mechanisms, the present findings do not characterize the full relationship between sleep and risk-taking behavior.

### 4.3. Future Directions

Future research should examine these associations using longitudinal, within-subjects designs, and in larger, more developmentally diverse samples spanning the adolescent transition. This period of development is marked by rapid changes in both sleep biology and the neural systems underlying decision-making. Additionally, examining the neural correlates of BELT performance using neuroimaging would allow for more direct testing of the prefrontal-salience network mechanisms proposed here, including whether sigma activity relates to prefrontal-striatal functional connectivity during task performance. Methodologically, future studies would benefit from multi-night recording protocols with an initial habituation night, high-density polysomnography enabling automated detection of slow and fast spindles across the scalp, objective verification of awakening times via wrist actigraphy and timed collection devices, and systematic assessment of hormonal variables including menstrual phase and contraceptive use. Such work would help to clarify whether the state-specific sleep associations observed in the present study are related to the developmental trajectories of prefrontal maturation known to occur across adolescence and young adulthood. Additionally, experimental manipulation of sleep architecture would provide a more direct test of whether changes in sleep produce corresponding changes in next-day risk-taking.

## 5. Conclusions

The present findings contribute to a growing literature linking specific features of sleep architecture to next-morning cognitive and behavioral functioning, and carry particular relevance for adolescent health. Adolescence represents a critical window during which risk-taking behavior peaks (Steinberg, 2008), sleep is most biologically and environmentally disrupted (Crowley et al., 2018), and neural systems underlying decision-making are still maturing (Caballero et al., 2016; Steinberg, 2008), a convergence that makes understanding the sleep-behavior relationship in this population both scientifically urgent and clinically meaningful. By identifying associations between REM sleep, sigma activity, morning cortisol and risk-taking behavior, this study underscores the value of a more nuanced account of how sleep architecture relates to next-day decision-making.

These findings carry tentative implications for settings in which young people’s sleep and decision-making intersect. Participants in this sample obtained an average of 5.81 h of sleep, consistent with broader evidence that adolescents and young adults are chronically sleep-restricted. While the correlational design precludes causal inference, the associations reported here suggest that the composition of sleep, not duration alone, may be relevant to next-morning decision-making. This is particularly salient for adolescent risk management, as the associations observed here concern decision-making under uncertainty and learning from negative outcomes. These are processes relevant to contexts in which young people make consequential choices with incomplete information, including early-morning driving, substance use, and health-related decisions. Behavioral risk-taking on balloon-analogue tasks has been linked to self-reported real-world risk behavior, including substance use, delinquency, and safety behaviors (Lejuez et al., 2002, 2003). To the extent that BELT performance indexes similar processes, habitual sleep may therefore warrant consideration as a modifiable factor in adolescent risk-reduction efforts. Evidence that delayed school start times increase sleep duration and may improve adolescent outcomes (e.g., Dunster et al., 2018; Marx et al., 2017) points to the value of intervention in this area. The present findings suggest a candidate mechanism through which such interventions might operate, and indicate that outcome measures capturing risk-related decision-making, rather than academic performance alone, may be informative.

## Figures and Tables

**Table 1:** Descriptive statistics for objective sleep architecture, behavioral task performance, and cortisol awakening response variables. Sleep architecture variables (*n* = 52) were derived from overnight Sleep Profiler recordings. Behavioral outcomes (*n* = 50) were assessed using the Balloon Emotional Learning Task (BELT). Cortisol awakening response (*n* = 36) is reported as the log-transformed area under the curve with respect to increase (AUCi) across the 45-min post-awakening sampling window. NREM = non-rapid eye movement; REM = rapid eye movement. Low = low explosion threshold (quick to pop); High = high explosion threshold.

	Minimum	Maximum	Mean	St. Dev
Total Sleep Time (hrs)	2.82	8.77	5.81	1.28
Sleep Efficiency (%)	33.17	96.44	84.05	12.78
Wake After Sleep Onset (min)	9.00	328.0	56.00	70.21
NREM1 (%)	0.96	26.22	6.76	5.30
NREM2 (%)	6.83	63.81	33.39	13.23
NREM3 (%)	16.93	80.47	40.15	14.73
REM (%)	0.00	33.55	19.70	8.75
BELT Total Points Earned	232	431	320.12	54.03
BELT Total Pops	4	31	14.88	5.95
BELT Total Pumps	275	613	447.92	87.99
BELT Total Pumps—Low	87	125	110.1	7.2
BELT Total Pumps—High	85	319	198.9	71.0
BELT Total Pumps—Variable	98	197	138.38	25.69
Log AUCi	1.72	33.12	13.48	6.94

## Data Availability

Data are made available on Open Science Framework: https://osf.io/rk3bq (accessed on 1 June 2026).

## Abbreviations

The following abbreviations are used in this manuscript:

PSQI	Pittsburgh Sleep Quality Index
REM	Rapid Eye Movement (sleep)
NREM	Non-Rapid Eye Movement (sleep)
HPA	Hypothalamic–Pituitary–Adrenal (axis)
CAR	Cortisol Awakening Response
ACTH	Adrenocorticotropic Hormone
EEG	Electroencephalographic/Electroencephalogram
LC-MS/MS	Liquid Chromatography Tandem Mass Spectrometry
SIRF	Shared Instrumentation and Research Facility
AUCi	Area Under the Curve with respect to increase
BELT	Balloon Emotional Learning Task
MEQ	Morningness-Eveningness Questionnaire
BIS/BAS	Behavioral Inhibition Scale/Behavioral Activation Scales
UPPS	Urgency, Premeditation, Perseverance, Sensation Seeking (Impulsive Behavior Scale)
N1/N2/N3 (NREM1/NREM2/NREM3)	Non-REM sleep stages 1, 2, 3
WASO	Wake After Sleep Onset
